# A Novel Homozygous Variant of *TMEM231* in a Case With Hypoplasia of the Cerebellar Vermis and Polydactyly

**DOI:** 10.3389/fped.2021.774575

**Published:** 2021-11-29

**Authors:** Tao Wang, Yu-Xing Liu, Fang-Mei Luo, Yi Dong, Ya-Li Li, Liang-Liang Fan

**Affiliations:** ^1^Departments of Reproductive Genetics, HeBei General Hospital, ShiJiaZhuang, China; ^2^Department of Cell Biology, School of Life Sciences, Central South University, Changsha, China; ^3^Hunan Key Laboratory of Animal Models for Human Disease, School of Life Sciences, Central South University, Changsha, China

**Keywords:** JBTS, MKS, *TMEM231*, mutation, homozygote, whole-exome sequencing

## Abstract

**Background:** Transmembrane protein 231 (TMEM231) is a component of the B9 complex that participates in the formation of the diffusion barrier between the cilia and plasma membrane. Mutations in *TMEM231* gene may contribute to the Joubert syndrome (JBTS) or Meckel–Gruber syndrome (MKS). However, reports on JBTS or MKS caused by *TMEM231* mutations are comparatively rare.

**Method:** We describe a Chinese fetus with unexplained hypoplasia of the cerebellar vermis and polydactyly, detected by ultrasound imaging. The fetus was primarily diagnosed with JBTS/MKS. The parents of this fetus were non-consanguineous and healthy. Whole-exome sequencing (WES) and bioinformatics strategies were employed to explore the genetic lesion of this family.

**Results:** An unknown missense variant (c.19C>T;p.R7W) of *TMEM231* gene was detected. The variant was predicted as pathogenic and was absent in our 200 healthy controls.

**Conclusion:** WES was employed to explore the genetic lesion of a fetus with unexplained hypoplasia of the cerebellar vermis and polydactyly. A novel variant in *TMEM231* gene was identified. Our study not only provided data for genetic counseling and prenatal diagnosis to this family but also broadened the spectrum of *TMEM231* mutations.

## Introduction

Ciliopathies are multiorgan system disorders caused by the defective cilium complex (1). B9 is a cilium complex at the transition zone (TZ) and protects the cilia as a privileged membrane domain (2, 3). The B9 complex includes more than 13 proteins. Genetic deficiency of any component of the B9 complex may lead to ciliopathy (4). Transmembrane protein 231 (TMEM231) is a part of the B9 complex that localizes at the base of the ciliary axoneme at the TZ (2). Mutations in *TMEM231* gene can contribute to the Joubert syndrome (JBTS, OMIM# 614970) or Meckel–Gruber syndrome (MKS, OMIM# 615397) (4, 5).

JBTS and MKS are rare but lethal ciliopathies with overlapping features (6). JBTS is an autosomal recessive (AR) disorder and is characterized by a distinctive mid-hindbrain malformation, oculomotor apraxia, abnormal breathing, intellectual impairment, and varying developmental delay. Some JBTS patients may also show retinopathy, renal cysts, and postaxial polydactyly (4). MKS is another AR disorder characterized by occipital encephalocele, polycystic kidneys, and polydactyly that is typically perinatally lethal (7). Pleiotropy is a common characteristic of ciliopathies; mutations in *TMEM231* gene with different degrees of severity may cause JBTS, MKS, or oral–facial–digital syndrome (OFDS). However, owing to the genetic heterogeneity and pleiotropy, the molecular basis of this variability remains largely unknown.

In this research, we described a Chinese fetus with unexplained hypoplasia of the cerebellar vermis and polydactyly detected by ultrasound imaging. The parents of this fetus were non-consanguineous and healthy. Employing whole-exome sequencing (WES) technology and bioinformatics strategies, we identified a novel homozygous variant in *TMEM231* gene.

## Materials and Methods

### Subjects and Ethical Approval

**Figure 1:**
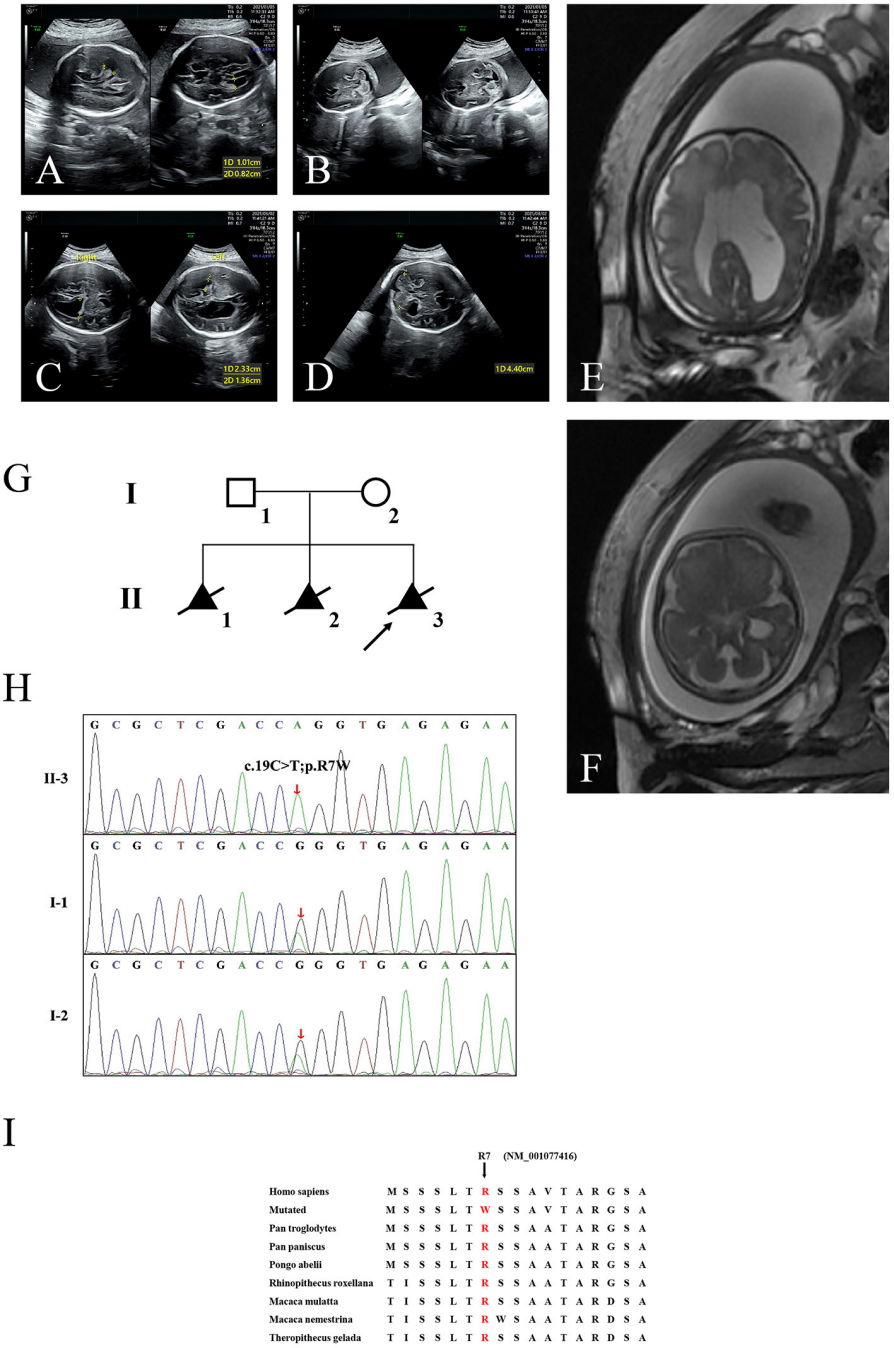
The clinic and sequencing data of this JBTS/MKS family. Ultrasound images show lateral ventricle **(A)** and cerebellum in transverse section **(B)** of the proband (II-3) at 22nd-week gestation. Lateral ventricle **(C)** and cerebellum **(D)** ultrasound images at 34th-week gestation. 3D FIESTA shows lateral ventricle **(E)** and cerebellum in transverse section **(F)** at 34th-week gestation. **(G)** The genealogy of this JBTS/MKS family. Squares indicate male family members; circles, female members; triangles, fetus; black symbols, the affected members; white symbols, unaffected members; arrow, proband. **(H)** Sanger DNA sequencing chromatogram detected a homozygosity mutation (c.19C>T;p.R7W) of *TMEM231* gene in the proband. **(I)** Alignment of multiple TMEM231 protein sequences across species. Letters in red show the R7 site (NM_001077416) are evolutionarily conserved. FIESTA, Fast Imaging Employing Steady-state Acquisition.

Here, we encountered a mother from the Hebei Province in China with a 22nd-week gestation. She visited our hospital owing to an antenatal ultrasound scan that revealed the vermis of the cerebellum was indistinct in the fetus. The ultrasound examination showed the right cerebral ventricle of the fetus was widened, and Dandy–Walker malformation was suspected (Figures 1A,B). Amniocentesis revealed normal karyotype (46, XY). The following antenatal ultrasound scan, at 34th-week gestation, revealed hydrocephalus, agenesis of the cerebellar vermis, and polydactyly in the fetus (Figures 1C–F), and the parents decided to terminate the pregnancy due to the fatal deformity. The mother was 30 years old and the father was 31 years old. They were non-consanguineous and healthy. Further investigation showed the parents come from one closed and small village. Family history survey showed that proband (II-3) was the product of the third pregnancy of this family, in which all three siblings were similarly affected (Figure 1G). As the ultrasound demonstrated hydrocephalus and cerebellar dysplasia in the fetuses, the first and the second pregnancies (II-1 and II-2) were terminated (Table 1). On the other hand, 200 healthy subjects as described in our previous study were enrolled in this study to exclude polymorphisms (8).

**Table 1:** Clinical data of three fetuses in this family.

**Subjects**	**II-1**	**II-2**	**II-3 (proband)**
Sex	M	F	M
Gestation (weeks)	32	29	34
Karyotype	46, XY	46, XX	46, XY
Hydrocephalus	+	+	+
Cerebellar dysplasia	+	+	+
Polydactyly	NA	NA	+
Termination of pregnancy	+	+	+

*F, female; M, male; NA, not available*.

This study was approved by the Review Board of the HeBei General Hospital, ShiJiaZhuang, China. Written informed consent was obtained from all adult participants and legal guardians of the minor participants.

### Whole-Exome Sequencing

Genomic DNA was extracted from peripheral blood lymphocytes of all the participants using the DNeasy Blood &Tissue Kit (Qiagen, Valencia, CA, USA.). The central part of the WES services and the necessary bioinformatics analyses were provided by the Novogene Bioinformatics Institute (Beijing, China). Exomes were captured using SureSelect Human All Exon V6 kits (Agilent, Santa Clara, CA, USA), and next-generation sequencing (NGS) was conducted with a HiSeq X-10 system (Illumina, San Diego, CA, USA). The strategies of data filtering refer to a previous study published by our laboratory group (9).

### Mutation Validation and Co-segregation Analysis

Sanger sequencing was used to validate the candidate variants identified in WES. Segregation analysis was performed in the family members of this study. Primer pairs were designed employing the PrimerQuest Tool IDT (http://sg.idtdna.com/Primerquest/Home/Index), and sequences of the polymerase chain reaction (PCR) products were determined using the ABI 3100 Genetic Analyzer (ABI, Foster City, CA).

### Bioinformatics Analysis

The Polyphen-2, SIFT, and MutationTaster programs were used to predict the effects of mutations on the function of the protein. Swiss-Model software (https://swissmodel.expasy.org/interactive) was used to identify the function of the mutation. Local hydrophobicity was predicted by ProtScale (https://web.expasy.org/protscale/). The conservation analysis was performed by comparing amino acid sequences among different species.

## Result

**Figure 2:**
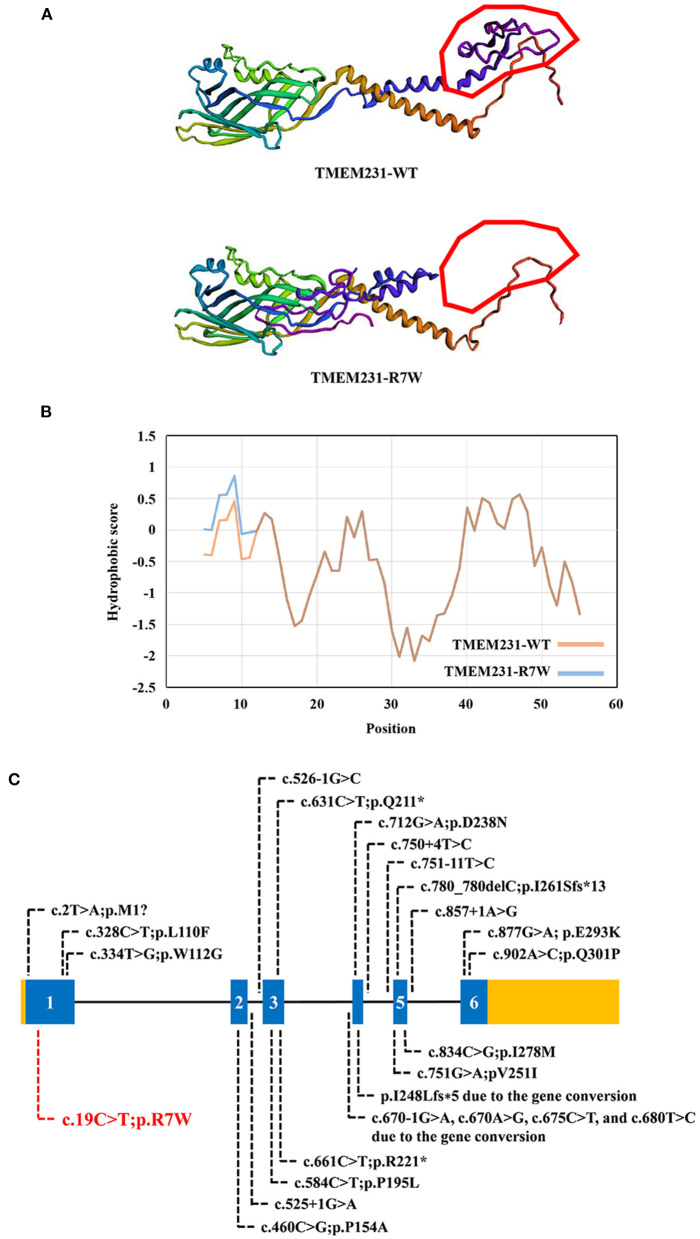
The bioinformatics analysis of mutations. **(A)** Structure prediction of the mutant protein. The wild-type TMEM231 (TMEM231-WT) protein structure and the p.R7W mutant TMEM231 (TMEM231-p.R7W) protein structure were predicted by SWISS-MODEL online software. **(B)** The Protscale online software predicts the hydrophobicity of wild-type and p.R7W mutant TMEM231 protein. The yellow curve shows the hydrophobicity score of each amino acid of wild-type TMEM231. The blue curve shows the p.R7W mutant TMEM231. **(C)** Schematic of *TMEM231* mutations identified in JBTS- and MKS-affected individuals. The *TMEM231* gene is showed, with all currently known *TMEM231* mutations (black letters) and novel mutation (red letters).

WES yielded 11.9 Gb of data with 99.1% coverage of the target region and 98.98% of the target covered over 10 ×. After data filtering, the variants were further filtered by ciliopathy-related genes as described in the previous study (10) (Supplementary Table 1). A set of six variants in five genes were detected and further analyzed. Information related to inheritance pattern, Online Mendelian Inheritance in Man (OMIM) clinical phenotypes (https://omim.org/), ToppGene gene function (11), and American College of Medical Genetics and Genomics (ACMG) classification (12) of these six variants are shown in Table 2. Sanger sequencing was performed in the family members (I-1, I-2, and II-3) and showed that a novel homozygous variant (c.19C>T;p.R7W) of the *TEME231* gene (NM_001077416) may underlie the genetic factor of this fetus with hypoplasia of the cerebellar vermis and polydactyly (Figure 1H). Each parent of this fetus carried an allele of this homozygous variant (Figure 1H). The (c.19C>T;p.R7W) variant targets exon 1 of the canonical isoform of *TMEM231* (NM_001077416), as well as the other predicted protein-coding isoforms (NM_001077418). In NM_001077416, the variant results in the substitution of arginine acid by tryptophan at code 7, while in NM_001077418, it leads to the substitution of proline acid by leucine at code 10 (c.29C>T;p.P10L). This variant was not present in our 200 healthy controls group. Alignment of TMEM231 (NM_001077416) amino acid sequences revealed that R7 is conserved in primates (Figure 1I). Furthermore, modeling of proteins before and after missense variant were performed by SWISS-MODEL software (https://swissmodel.expasy.org/) and revealed that the missense variant at R7W may lead to the change of TMEM231 protein structure as marked by the red frame in the figure (Figure 2A). ProtScale software (https://web.expasy.org/protscale/) analyses revealed that the R7W variant raised local hydrophobicity compared with wild-type (WT) protein (Figure 2B).

**Table 2:** Variants identified by WES in this family.

**Gene**	**Transcript variant**	**Protein variant**	**SIFT**	**Polyphen-2**	**Mutationtaster**	**GnomAD**	**OMIM clinical phenotype**	**ToppGene function**	**American College of Medical Genetics classification**	**Carrier**
*TMEM231*	NM_001077416 c.19C>T	p.R7W	D	D	D	–	AR, Joubert syndrome or Meckel syndrome	Ciliary transition zone	PM2; PP3	*I-1*; *I-2*; II-3
*NPHP4*	NM_015102 c.2849G>A	p.R950Q	T	D	D	0.000120272	AR, Nephronophthisis	Structural molecule activity	PM2	*I-2*; II-3
*EVC2*	NM_147127 c.1882G>A	p.E628K	T	B	D	0.000918672	AR, Ellis-van Creveld syndrome	Ciliary membrane	PM5; BS2	*I-1*; II-3
*EVC2*	NM_147127 c.1341C>T	–	–	–	D	0.000377824	AR, Ellis-van Creveld syndrome	Ciliary membrane	PM2; BS2	*I-1*; II-3
*FLNA*	NM_001456 c.2472C>T	–	–	–	D	0.000049715	XLR, Congenital short bowel syndrome	Fc-gamma receptor I complex binding	PM2	*I-1*; II-3
*GLIS2*	NM_032575 c.1010C>T	p.P337L	D	D	D	0.00188042	AR, Nephronophthisis	CTNNB1 binding	PP3; BS1	*I-2*; II-3

*B, begin; D, disease-causing; P, polymorphism; T, tolerated; AD, autosomal dominant; AR, autosomal recessive; XLD, X-linked dominant inheritance; BS, strong benign; PP, pathogenic supporting; PM, pathogenic moderate. Italic text represents healthy family members*.

## Discussion

In this study, we reported a Chinese fetus with unexplained hypoplasia of the cerebellar vermis and polydactyly detected by ultrasound imaging. The fetus was primarily diagnosed with JBTS/MKS. The parents of this fetus were non-consanguineous and healthy. A new homozygous variant (c.19C>T, p.R7W) of *TMEM231* gene was detected by employing WES in combination with ciliopathy-related gene-filtering. Sanger sequencing confirmed that this fetus (II-3) harbored the homozygous missense variant in *TMEM231*, and each parent of this fetus carried an allele of this homozygous variant. DNA from the first (II-1) and the second fetal demises (II-2) were not available. Given that both the parents carried the *TMEM231* c.19C>T;p.R7W heterozygous variant and all three siblings had similar phenotypes, we suspected that their fetuses (II-1 and II-2) with hydrocephalus and cerebellar dysplasia also carried this homozygous variant.

JBTS and MKS are rare ciliopathies. JBTS patients manifest a characteristic “molar tooth sign” on brain imaging in conjunction with retinopathy, nephropathy, and polydactyly. Although some individuals with JBTS die in infancy, most of the patients survive with variable developmental outcomes. MKS is a much more deadly perinatal syndrome. As the severe part of the ciliopathy phenotypic spectrum, MKS-affected individuals present with polycystic kidneys, occipital encephalocele, and polydactyly (13). To date, mutations in more than 27 genes are associated with JBTS, and pathogenic variants in at least 17 genes have been identified in MKS patients. Importantly, mutations in 12 of these genes, including *TMEM231, TMEM67*, and TEME237, are reported to cause both conditions (13, 14). As JBTS and MKS share overlapping features and are genetically heterogeneous, some laboratories routinely deploy a gene panel diagnostic approach that examines all JBTS- or MKS- associated disease genes, irrespective of which of the two diagnostic categories the clinical features suggest (13). Additionally, *TMEM231* mutations have also been reported in OFDS patients. OFDS are rare disorders characterized by facial, oral, and digital abnormalities associated with a broad range of additional features. TMEM231, initially implicated in JBTS and MKS, also caused unclassified OFDS, with cerebellar hypoplasia, severe microcephaly, or polycystic kidney disease. The OFDS phenotype was clinically heterogeneous, and recent researches have confirmed the clinical and genetic overlap between OFDS and other ciliopathies, such as JBTS and MKS (15). A more comprehensive review of previously reported cases with *TMEM231* variants is presented in Table 3 and may help to establish a genotype–phenotype correlation (18–21). Consistent with the previous study, the present study was also based on a non-consanguineous family. The proband was a product of the third pregnancy of this family and was primarily diagnosed as JBTS/MKS. A homozygote variant (c.19C>T;p.R7W) in *TMEM231* was identified as a possible cause of the genetic lesion in this family. Besides, Dandy–Walker malformation is also one of the symptoms associated with MKS (22). The same was observed in the proband of the present study.

**Table 3:** The summary of reported patients with *TMEM231* variant.

**Patient reported**	**Our patient**	**Srour et al. (4)**	**Shaheen et al. (5)**	**Shaheen et al. (14)**	**Braun et al. (16)**	**Maglic et al. (6)**	**Maglic et al. (6)**
Variation	p.R7W	p.M1?; p.D238N	p.V251I; p.Q301P	c.751-11T>C	p.L110F; p.P154A	p.D283N; gene conversion	p.W112G; gene conversion
PMID	–	23012439	23349226	27894351	26489029	27449316	27449316
Sex	M	F	F	M	-	M	F
Age	TOP	4 years	–	2 years	13 years	4 years	–
Zygosity	Hom	CHet	CHet	Hom	CHet	CHet	CHet
Type	JBTS/MKS	JBTS	MKS	JBTS	OFD	JBTS/MKS	MKS
Brain structure abnormalies	Agenesis of the cerebellar vermis, DWN	MTS	Occipital encephalocele	MTS; stenogyria/microgyria in the posterior and medial occipital cortex	Agenesis of the cerebellar vermis, DWN	MTS	Occipital encephalocele
Limb abnormality	Polydactyly	Postaxial polydactyly and syndactyly of the right foot	Polydactyly	Polydactyly	Polydactyly	–	Polydactyly
Urogenital anomalies	–	CK	Polycystic kidney	Renal failure	End stage renal disease	-	Enlarged polycystic kidneys
Developmental delay	–	+	–	+	–	+	–
Oculomotor apraxia	–	+	–	+	+	–	–
Breathing abnormality	–	+	–	+	–	–	–
Retinal involvement	–	+	–	+	–	+	–
Facial features	–	–	–	–	–	–	–
Others	–	–	–	–	Lingual hamartomas, intellectual disability	–	–
**Patient reported**	**Bruel et al**. **(****5****)**	**Roberson et al**. **(****17****)**	**Roberson et al**. **(****17****)**	**Roberson et al**. **(****17****)**	**Roberson et al**. **(****17****)**	**Roberson et al**. **(****2****)**	**Roberson et al**. **(****17****)**
Variation	p.P195L; p.P154A	p.P154A	p.P154A; p.Q211*	p.I261Sfs*13, c.750+4A>G	c.750+4A>G	c.750+4A>G	p.P154A; c.750+4A>G
PMID	28289185	25869670	25869670	25869670	25869670	25869670	25869670
Sex	F	-	–	–	–	–	–
Age	42 year	40 weeks	20 weeks	13 weeks	14 weeks	22 weeks	25 weeks
Zygosity	CHet	Hom	CHet	CHet	Hom	Hom	CHet
type	OFD	MKS	MKS	MKS	MKS	MKS	MKS
Brain structure abnormalies	Agenesis of the cerebellar vermis, DWN	DWN, hydrocephalus	Agenesis of the cerebellar vermis, DWN	Anencephaly	Meningoencephalocele, brain malformation	Meningoencephalocele	Hydrocephalus, holoprosencephaly
Limb abnormality	Post-axial polydactyly	Polydactyly	Polydactyly	Polydactyly	Polydactyly	Polydactyly	Polydactyly
urogenital anomalies	-	CK	CK	CK	CK, epididymal cysts	CK	CK
Developmental delay	–	–	–	–	–	–	–
Oculomotor apraxia	–	–	–	–	–	–	–
Breathing abnormality	–	–	–	–	–	–	–
Retinal involvement	–	–	–	–	–	–	–
Facial features	Clef lip, CP, hypertelorism, micro/retrognathia	–	–	–	CP	–	–
Others	Hypoplasia 12th pair of rib	Hepatic portal fibrosis	Hepatic portal fibrosis	–	Hepatic portal fibrosis, single umbilical artery	Hepatic portal fibrosis, Pancreatic fibrosis	Hepatic portal fibrosis, Pancreatic fibrosis
**Patient reported**	**Roberson et al**. **(****17****)**	**Kroes et al**. **(****18****)**	**Watson et al**. **(****13****)**	**Nicolas-Jilwan et al**. **(****19****)**	**Li et al**. **(****20****)**
Variation	c.526-1G>C; p.A245P	p.I278M	c.857+1A>G; gene conversion	c.751-11T>C	p.R221, c.525+1G>A
PMID	25869670	25920555	31663672	30617574	32386258
Sex	–	F	M	M	–
Age	17 weeks	4 years	13 weeks	3 months	–
Zygosity	CHet	Hom	CHet	Hom	CHet
Type	MKS	JBTS	JBTS/MKS	JBTS	MKS
Brain structure abnormalies	Meningoencephalocele	–	Occipital encephalocoele	Agenesis of the cerebellar vermis, MTS, occipicervical encephalocele, ventriculomegaly	DWM, hydrocephalus, encephalocele
Limb abnormality	Polydactyly	Unilateral postaxial polydactyly	Postaxial polydactyly of both hands, bilateral talipes equinovarus	Bilateral postaxial polydactyly	Bilateral talipes equinovarus, angulation of bilateral radius
Urogenital anomalies	CK, epididymal cysts	–	Enlarged multicystic dysplastic kidneys	–	Bilateral multicystic kidney dysplasia, absent of bladder
Developmental delay	–	+	–	–	–
Oculomotor apraxia	–	+	–	+	–
Breathing abnormality	–	–	–	+	–
Retinal involvement	–	–	–	–	–
Facial features	CP	–	Small low set ears	–	–
Others	Single umbilical artery	–	Hepatic ductal plate malformation	Seizures	Skeletal abnormalities

*CHet, compound heterozygous; CK, cystic kidneys; CP, cleft palate; DWN, Dandy–Walker malformation; F, female; Hom, homozygous; JBTS, Joubert syndrome; M, male; MKS, Meckel-Gruber syndrome; MTS, molar tooth sign; OFD, oral-facial-digital syndrome*.

Tmem231 is a 36-kD two-pass transmembrane protein that locates at the base of the ciliary axoneme at the TZ (2). Tmem231 has a role in mammalian development (23). In C57BL/6 mice, *Tmem231*^−/−^ embryos die before birth with distinct features of ciliopathy, including abrogated hedgehog signaling and polydactyly (2). *Tmem231* mutant mouse embryos also exhibit ciliopathy hallmarks such as polydactyly, microphthalmia, and dorsalization of the neural tube (17). As a part of the B9 complex, TMEM231 is crucial for the constitution and functions of the cilia and physically interacts with many JBTS- or MKS-related genes (2, 17). Therefore, mutations in *TMEM231* may lead to JBTS/MKS. In our research, we detected a homozygous variant (c.19C>T;p.R7W) of *TMEM231* in a fetus with unexplained hypoplasia of the cerebellar vermis and polydactyly. This missense variant (p. R7W) locates in the exon 1 of *TMEM231* gene that alters the arginine codon at position 7 to a tryptophan codon. As predicted by the SWISS-MODEL software, the missense variant at R7W may lead to the change of TMEM231 protein structure (Figure 2A). ProtScale software analyses revealed that the local hydrophobicity at and near the altered amino acid was increased compared with the WT TMEM231 protein (Figure 2B). In this case, the structural change might affect the transmembrane and localization of TMEM231 protein. And increased hydrophobicity at the R7W variant site might affect protein–protein interactions. No other TMEM231 coding or splicing variants were detected in the exome of this fetus. Furthermore, gene conversion in *TEME231* may also lead to JBTS/MKS. As WES does not apply to detecting large deletions, ciliopathy patients who were only detected one heterozygous *TMEM231* variant should be further examined (6).

By far, ~20 variants of *TMEM231*, including two gene conversions, have been identified in JBTS-, MKS-, and OFDS-affected individuals (Figure 2C). Although the pathogenic mechanism involved requires further investigation, our findings offer more evidence that *TMEM231* gene variation is significant in JBTS/MKS. Notably, the variant (c.19C>T;p.R7W) detected in this research has not been published earlier and, therefore, is considered novel.

## Conclusion

We used WES to explore the genetic lesion in a Chinese fetus with unexplained hypoplasia of the cerebellar vermis and polydactyly. A novel homozygous variant (c.19C>T;p.R7W) of *TMEM231* was detected. Our study not only provided data for genetic counseling and prenatal diagnosis to this family but also broadened the spectrum of *TMEM231* mutations.

## Data Availability Statement

The datasets presented in this study can be found in online repositories. The names of the repository/repositories and accession number(s) can be found at: NCBI BioSample; PRJNA773747.

## Ethics Statement

The studies involving human participants were reviewed and approved by the Review Board of the HeBei General Hospital, ShiJiaZhuang, China. Written informed consent to participate in this study was provided by the participants' legal guardian/next of kin. Written informed consent was obtained from the individual(s), and minor(s)' legal guardian/next of kin, for the publication of any potentially identifiable images or data included in this article.

## Author Contributions

TW and Y-XL enrolled the family members. F-ML performed DNA isolation and Sanger sequencing. YD performed genetic analysis and bioinformatics analysis. Y-XL and L-LF wrote the manuscript. Y-LL and L-LF supported the project. All authors reviewed the manuscript.

## Funding

This study was supported by National Natural Science Foundation of China (82000427), National Natural Science Foundation of Hunan province (2020JJ5785), the Fundamental Research Funds for the Central Universities of Central South University (2021zzts0079), Hunan Provincial Innovation Foundation for Postgraduate (CX20210177), and the clinical medical personnel training program of Hebei Provincial Health Commission (Y-LL).

## Conflict of Interest

The authors declare that the research was conducted in the absence of any commercial or financial relationships that could be construed as a potential conflict of interest.

## Publisher's Note

All claims expressed in this article are solely those of the authors and do not necessarily represent those of their affiliated organizations, or those of the publisher, the editors and the reviewers. Any product that may be evaluated in this article, or claim that may be made by its manufacturer, is not guaranteed or endorsed by the publisher.
