# Supplementary material for: A Novel Homozygous Variant of TMEM231 in a Case With Hypoplasia of the Cerebellar Vermis and Polydactyly
Source: Front Pediatr. 2021 Nov 29;9:774575. doi: 10.3389/fped.2021.774575 (PMC8666876; doi:10.3389/fped.2021.774575)
Supplement: Supplementary file 1 [file Table_1.docx]

Supplementary Table 1. Panel with 115 ciliopathy related genes.

| ***AHI1*** | ***CD96*** | ***FRAS1*** | ***KIAA0586*** | ***Rab23*** | ***TTC8*** |
| --- | --- | --- | --- | --- | --- |
| ***AIPL1*** | ***CEP83*** | ***GDF6*** | ***KIF7*** | ***RD3*** | ***TTC21B*** |
| ***ALMS1*** | ***CEP164*** | ***GlI3*** | ***LCA5*** | ***RDH12*** | ***TULP1*** |
| ***ANKS6*** | ***CEP290*** | ***GLIS2*** | ***LRAT*** | ***RFX3*** | ***WDPCP*** |
| ***ATXN10*** | ***CEP41*** | ***GJA1*** | ***LZTFL1*** | ***RFX7*** | ***TMEM67*** |
| ***ARL13B*** | ***CRB1*** | ***GRIP1*** | ***MERTK*** | ***ROR2*** | ***TMEM138*** |
| ***ARL6*** | ***CRX*** | ***GUCY2D*** | ***MKKS*** | ***RPE65*** | ***TMEM216*** |
| ***B9D1*** | ***CSPP1*** | ***IFT52*** | ***MKS1*** | ***RPGRIP1*** | ***TMEM231*** |
| ***B9D2*** | ***DDX59*** | ***IFT57*** | ***NEK1*** | ***RPGRIP1L*** | ***TMEM237*** |
| ***BBS1*** | ***DYNC2H1*** | ***IFT80*** | ***NEK8*** | ***SCLT1*** | ***TRIM32*** |
| ***BBS10*** | ***DNAH2*** | ***IFT88*** | ***NMNAT1*** | ***SDCCAG8*** | ***WDR19*** |
| ***BBS12*** | ***EVC*** | ***IFT122*** | ***NPHP1*** | ***SLC41A1*** | ***WDR34*** |
| ***BBS2*** | ***EVC2*** | ***IFT140*** | ***NPHP3*** | ***SLSN3*** | ***WDR35*** |
| ***BBS4*** | ***EFNB1*** | ***IFT172*** | ***NPHP4*** | ***SPATA17*** | ***WDR60*** |
| ***BBS5*** | ***EXOC4*** | ***IMPDH1*** | ***OFD1*** | ***TBC1D32*** | ***ZNF423*** |
| ***BBS7*** | ***FREM2*** | ***INPP5E*** | ***OTX2*** | ***TCF4*** |  |
| ***C2CD3*** | ***FLNA*** | ***INTU*** | ***PKD1*** | ***TCTN1*** |  |
| ***C5ORF42*** | ***FUZ*** | ***INVS*** | ***PKD2*** | ***TCTN2*** |  |
| ***CC2D2A*** | ***GANAB*** | ***IQCB1*** | ***PKHD1*** | ***TCTN3*** |  |
| ***CCDC28B*** | ***FGFR1*** | ***KCNJ13*** | ***PTHB1*** | ***TFAP2A*** |  |
